# Secure bioinformatics: privacy-preserving federated analytics using homomorphic encryption

**DOI:** 10.1093/bioinformatics/btag081

**Published:** 2026-02-20

**Authors:** Weizhuang Zhou, Chao Jin, Zexi Yao, Meenatchi Sundaram Muthu Selva Annamalai, Yu En Chan, Sreejith Kumar Ashish Jith, Xiaoxia Deng, Fook Mun Chan, Kok Leong Foong, Rayden Chua Ming Hong, Kok Wai Wong, Roger Foo Sik Yin, Carolyn S P Lam, Arthur Mark Richards, Weng Khong Lim, Jonathan Yap, Khung Keong Yeo, Boon Ooi Patrick Tan, Neerja Karnani, Pavitra Krishnaswamy, Sebastian Maurer-Stroh, Khin Mi Mi Aung

**Affiliations:** Institute for Infocomm Research, Agency for Science, Technology and Research (A*STAR), Singapore 138632, Singapore; Institute for Infocomm Research, Agency for Science, Technology and Research (A*STAR), Singapore 138632, Singapore; Institute for Infocomm Research, Agency for Science, Technology and Research (A*STAR), Singapore 138632, Singapore; Institute for Infocomm Research, Agency for Science, Technology and Research (A*STAR), Singapore 138632, Singapore; Institute for Infocomm Research, Agency for Science, Technology and Research (A*STAR), Singapore 138632, Singapore; Institute for Infocomm Research, Agency for Science, Technology and Research (A*STAR), Singapore 138632, Singapore; Institute for Infocomm Research, Agency for Science, Technology and Research (A*STAR), Singapore 138632, Singapore; Institute for Infocomm Research, Agency for Science, Technology and Research (A*STAR), Singapore 138632, Singapore; Institute for Infocomm Research, Agency for Science, Technology and Research (A*STAR), Singapore 138632, Singapore; Institute for Infocomm Research, Agency for Science, Technology and Research (A*STAR), Singapore 138632, Singapore; Institute for Infocomm Research, Agency for Science, Technology and Research (A*STAR), Singapore 138632, Singapore; Department of Cardiology, National Heart Centre Singapore, Singapore 169609, Singapore; Yong Loo Lin School of Medicine, NUHS Tower Block, Singapore 119228, Singapore; Department of Cardiology, National Heart Centre Singapore, Singapore 169609, Singapore; Duke-NUS Medical School, Singapore 169857, Singapore; Yong Loo Lin School of Medicine, NUHS Tower Block, Singapore 119228, Singapore; Genome Institute of Singapore, Agency for Science, Technology and Research (A*STAR), Singapore 138672, Singapore; SingHealth Duke-NUS Genomic Medicine Centre, Singapore 168582, Singapore; SingHealth Duke-NUS Institute of Precision Medicine, Singapore 169609, Singapore; Cancer & Stem Cell Biology Program, Duke-National University of Singapore Medical School, Singapore 169857, Singapore; Department of Cardiology, National Heart Centre Singapore, Singapore 169609, Singapore; Duke-NUS Medical School, Singapore 169857, Singapore; Department of Cardiology, National Heart Centre Singapore, Singapore 169609, Singapore; Duke-NUS Medical School, Singapore 169857, Singapore; Genome Institute of Singapore, Agency for Science, Technology and Research (A*STAR), Singapore 138672, Singapore; Precision Health Research, Singapore 139234, Singapore; Yong Loo Lin School of Medicine, NUHS Tower Block, Singapore 119228, Singapore; Bioinformatics Institute, Agency for Science, Technology and Research (A*STAR), Singapore 138671, Singapore; Institute for Human Development and Potential, Agency for Science, Technology and Research (A*STAR), Brenner Centre for Molecular Medicine, Singapore 117609, Singapore; Department of Biochemistry, National University of Singapore, Singapore 117558, Singapore; Institute for Infocomm Research, Agency for Science, Technology and Research (A*STAR), Singapore 138632, Singapore; Yong Loo Lin School of Medicine, NUHS Tower Block, Singapore 119228, Singapore; Bioinformatics Institute, Agency for Science, Technology and Research (A*STAR), Singapore 138671, Singapore; Department of Biological Sciences, National University of Singapore, Singapore 117558, Singapore; Institute for Infocomm Research, Agency for Science, Technology and Research (A*STAR), Singapore 138632, Singapore

## Abstract

**Motivation:**

Large-scale bioinformatics analyses increasingly require collaboration across multiple cohorts and institutions, yet existing workflows often rely on data co-localization, which is slow, difficult to scale, and raises privacy concerns. We present a privacy-preserving federated analytics framework that enables secure statistical analysis across distributed datasets without transferring raw data, by performing all computations on encrypted data via cryptographic methods.

**Results:**

We evaluate the framework by validating polygenic risk scores and conducting meta-analyses on two real-world cohorts. The proposed solution achieves over 99.9% accuracy relative to plaintext analyses, while maintaining scalable runtime performance with increasing data size and number of participating sites. These results demonstrate the feasibility of secure federated analytics for practical bioinformatics applications involving sensitive data.

## Introduction

Modern bioinformatics research is increasingly performed across different data modalities, ranging from molecular and cellular ‘-omics’ data, to lifestyle and digital phenotypes, and to traditional health and medical records. This trend is fueled in part by the exponential growth of data collected by various lifestyle and wellness applications, direct-to-consumer genome sequencing and digitalization of healthcare records globally. A significant body of literature built on such datasets have demonstrated that large-scale multi-modal data analysis can provide invaluable biological insights (Fairley *et al.* 2020, del Pozo Cruz *et al.* 2022, Feng *et al.* 2023). However, obtaining centralized access to multiple datasets for research purposes is typically a herculean effort in and of itself, not least because datasets are often siloed within different institutions or organizations that each have their own data privacy considerations and sensitivities relating to transfer of data Bahmani *et al.* (2021). Commonly, separate data access agreements have to be signed with each data owner for a pre-specified use case before the datasets can be transferred to a central location, and only after that can downstream analysis begin. The approval process is typically lengthy, and introduces significant inefficiencies in scientific research.

**Figure 1:**
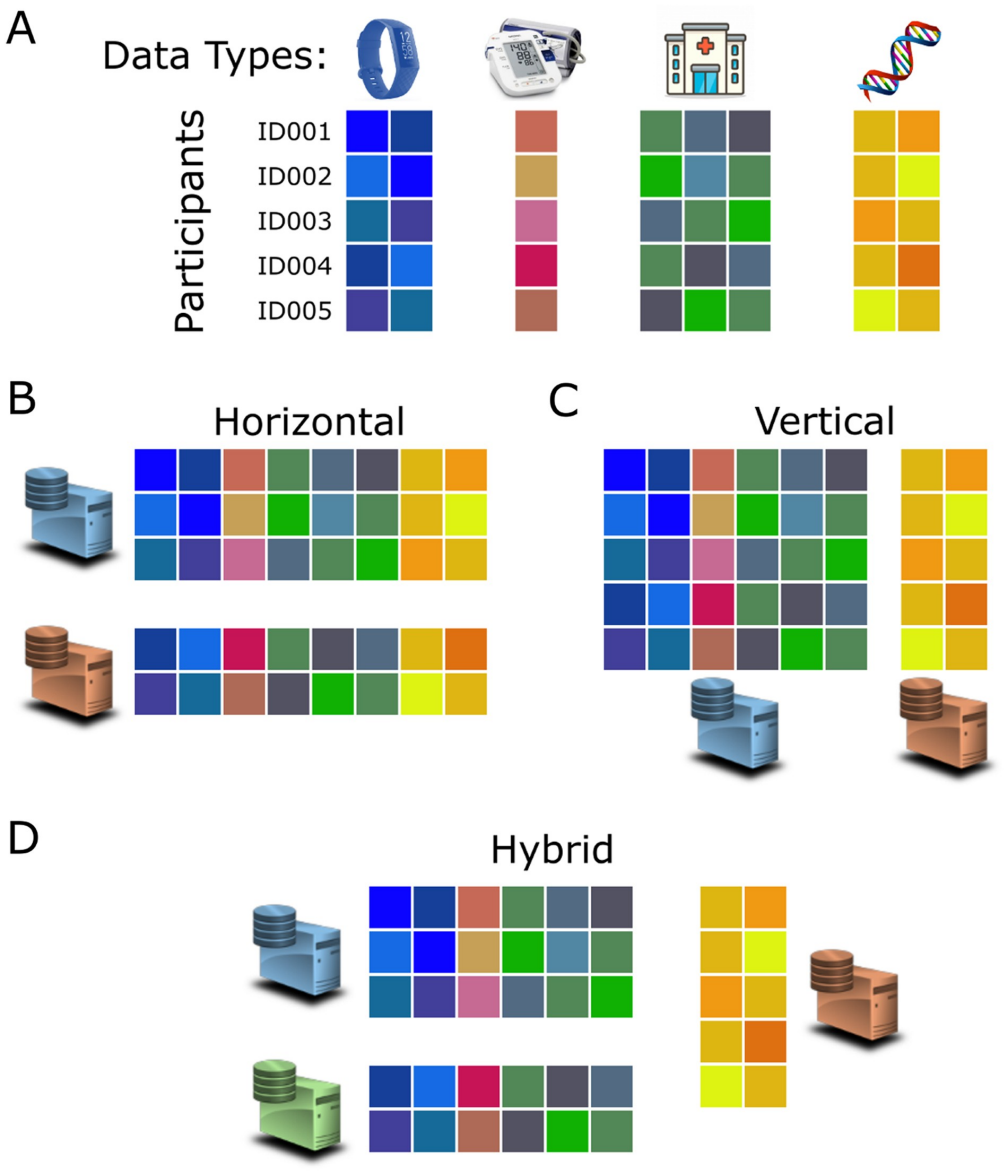
Multimodal data types and different partitioning schemes in the real world.

One promising solution to data privacy challenges in collaborative bioinformatics research is Homomorphic Encryption (HE), which enables computations to be performed directly on encrypted data without requiring decryption. However, despite its strong privacy guarantees, the widespread adoption of HE in biomedical applications has been limited, primarily due to its high computational overhead when applied to large-scale, high-dimensional *omics* datasets. Nevertheless, HE has been successfully applied to genomic analyses such as genome-wide association studies (GWAS) (Wang *et al.* 2016, Kim *et al.* 2020, Sim *et al.* 2020). In addition, federated analytics under horizontal data partitioning, where each site holds complete data modalities for different participants (Fig. 1B), has been demonstrated using multi-party homomorphic encryption (MHE) (Froelicher *et al.* 2021). More recently, MHE has been extended to support vertical data partitioning (Fig. 1C) (Geva *et al.* 2023) as well. In practice, real-world datasets often follow a hybrid of horizontal and vertical partitioning (Fig. 1D), which introduces additional challenges for secure collaborative analytics. For example, in collaborative polygenic risk score (PGS) evaluation across multiple cohorts, clinical phenotypes may be held separately by each healthcare institution, while shared genomic data for overlapping participants are stored at a third site, such as a sequencing provider.

On the other hand, secure multiparty computation (MPC) frameworks, such as MP-SPDZ (Keller 2020), enable collaborative computation over secretly shared data without revealing individual inputs. While MPC offers greater flexibility for general computations, it often incurs substantial communication overhead, particularly when operating on large batches of data.

In this work, we present our secure federated analytics platform that enables efficient and privacy-preserving collaborative bioinformatics research across multiple data sites. Our approach integrates MHE and MPC protocols to efficiently support the diverse mathematical operations required in typical analytics queries. We demonstrate the flexibility of the proposed framework by evaluating polygenic risk scores across heterogeneous data modalities. Specifically, we perform privacy-preserving statistical analyses on two real-world study cohorts, SingHEART (National Heart Centre Singapore 2016) and ATTRaCT, and evaluate both the accuracy of the analytical results and the associated runtime performance, demonstrating the practical feasibility of secure federated analytics for biomedical data.

## Materials and methods

### Evaluation of polygenic risk scores for hypertension across multiple cohorts

Three polygenic risk scores for hypertension have recently been developed using data from the UK Biobank (Sudlow *et al.* 2015, Bycroft *et al.* 2018): PGS000706 (Sinnott-Armstrong *et al.* 2021), PGS000957 and PGS000958 (Tanigawa *et al.* 2022). It is of clinical interest to understand how indicative these PGS would be for systolic blood pressure levels found in a different population such as Singapore, where the major ethnicity is Asian rather than European.

We consider its application on two study cohorts from Singapore: (i) the SingHEART study (NCT02791152) which consisted of relatively healthy volunteers, and (ii) the Asian Network for Translational Research and Cardiovascular Trials (ATTRaCT) study which consisted of patients with a history of cardiovascular disease. The control groups in each study were defined as participants without a medical history of hypertension and had normal blood pressure levels at the time of enrollment (systolic blood pressure < 130 mmHg and diastolic blood pressure < 85 mmHg). In contrast, case groups were defined as participants with either a medical history of hypertension or elevated blood pressure (systolic blood pressure ≥ 130 mmHg or diastolic blood pressure ≥ 85 mmHg) at the time of enrollment.

The definitions for the respective polygenic risk scores were downloaded from the PGS Catalog (Lambert *et al.* 2021). Similar to Ni *et al.* (2021), we standardized the polygenic risk scores within each cohort using ${\hat{s}}_{i}=\frac{s_{i}-\mu_{\mathrm{control}}}{\sigma_{\mathrm{control}}}$, where $s_{i}$ is the polygenic score of participant *i*, $\mu_{\mathrm{control}}$ and $\sigma_{\mathrm{control}}$ are the mean and standard deviation respectively of the scores of the cohort’s control group. For each cohort, we then grouped the participants based on quintiles of the standardized scores.

#### Demonstration of statistical testing

We performed quantitative evaluations to compare the mean systolic blood pressure of the five groups:

1. Comparison of the means of all five groups (ANOVA)
2. Comparison of the means of the highest genetic risk group to the lowest genetic risk group, i.e. between the fifth and first quintile (*t*-test)
3. Comparison of the means of the highest genetic risk group to the average genetic risk group, i.e. between the fifth and third quintile (*t*-test)

#### Demonstration of meta-analysis of standardized mean differences

Beyond testing for statistical differences in systolic blood pressure between the different genomic risk groups, we also demonstrate the ability of our secure platform in performing a meta-analysis across two cohorts, which is more commonly performed in research than a simplistic pooling of underlying datasets to form a single combined one. Specifically, we compared the systolic blood pressure of cases within each cohort between the lowest genomic risk group (PGS in the first quintile) and the highest genomic risk group (PGS in the fifth quintile), and meta-analyzed the effect size across the two cohorts (SingHEART and ATTRaCT) using a fixed-effect inverse-variance weighted model. We repeated this for each of the three polygenic risk scores above.

### Primer on privacy-preserving tools

Our secure solution leverages three main privacy-preserving components, which we describe in detail here.

#### Multi-party homomorphic encryption (MHE)

Homomorphic encryption (HE) is a unique type of encryption scheme that allows computations on encrypted data (ciphertext) without necessitating the decryption key. The result of the computation, once decrypted, is identical to the outcome obtained from performing regular arithmetic operations on the unencrypted data (i.e. plain-text data). Fully Homomorphic Encryption supports both homomorphic addition and multiplication on ciphertexts (Gentry 2009), and provides the *bootstrapping* primitive, which can reduce noise in ciphertext and enable theoretically arbitrary computations on ciphertext. In this work, we focus on a *multi-party* variant of the HE scheme (Mouchet *et al.* 2021). Under multi-party setting, each party samples his own part of the private key, and all the parts together constitute the additive secret-sharing form of the private key. After that, all the parties collaboratively generate the common public keys for encryption and ciphertext computation. The decryption process is also done collaboratively by all the parties, with each party doing a partial decryption on the ciphertext with his own share of the private key.

#### Secure multi-party computation (MPC)

Secure multi-party computation (MPC) enables secure computation among multiple parties who jointly evaluate a function while keeping their private inputs undisclosed. Yao’s Garbled Circuit (Yao 1982) was the first secure two-party computation solution based on encrypted Boolean Circuits. Generic MPC among more than two parties typically employs secret-sharing techniques such as additive secret-sharing or threshold secret-sharing (Shamir 1979) for input data.

#### Conversion between MHE and MPC

MHE is generally more communication-efficient, while MPC is more computation-efficient. It is possible to implement the HE2MPC and MPC2HE primitives to switch the private data between MHE encrypted form and MPC secret-shared form (Mouchet *et al.* 2021), in order to leverage both forms of secure computation to achieve optimal performance.

#### Private set intersection (PSI)

Private set intersection (PSI) is a key building block in federated data analytics. When datasets are distributed across different sites, it is necessary to cross-link the different data modalities for each participant (Fig. 1) before downstream analysis can be done meaningfully. PSI is a set of protocols that will perform this mapping of data in a secure manner, requiring only data owners to reveal the subset of participants common in all involved datasets. Formally, for a two-party PSI, one party (e.g. the request server) has a set *X*, and the other party (e.g. the client) has another set *Y*. The goal is for the client (or the server) to find out the items that both sets have in common (X∩Y) without learning about any other items in set *X* (or *Y*). In a similar vein, PSI can naturally be extended to more than two parties as well.

### Secure federated statistical analysis

**Figure 2:**
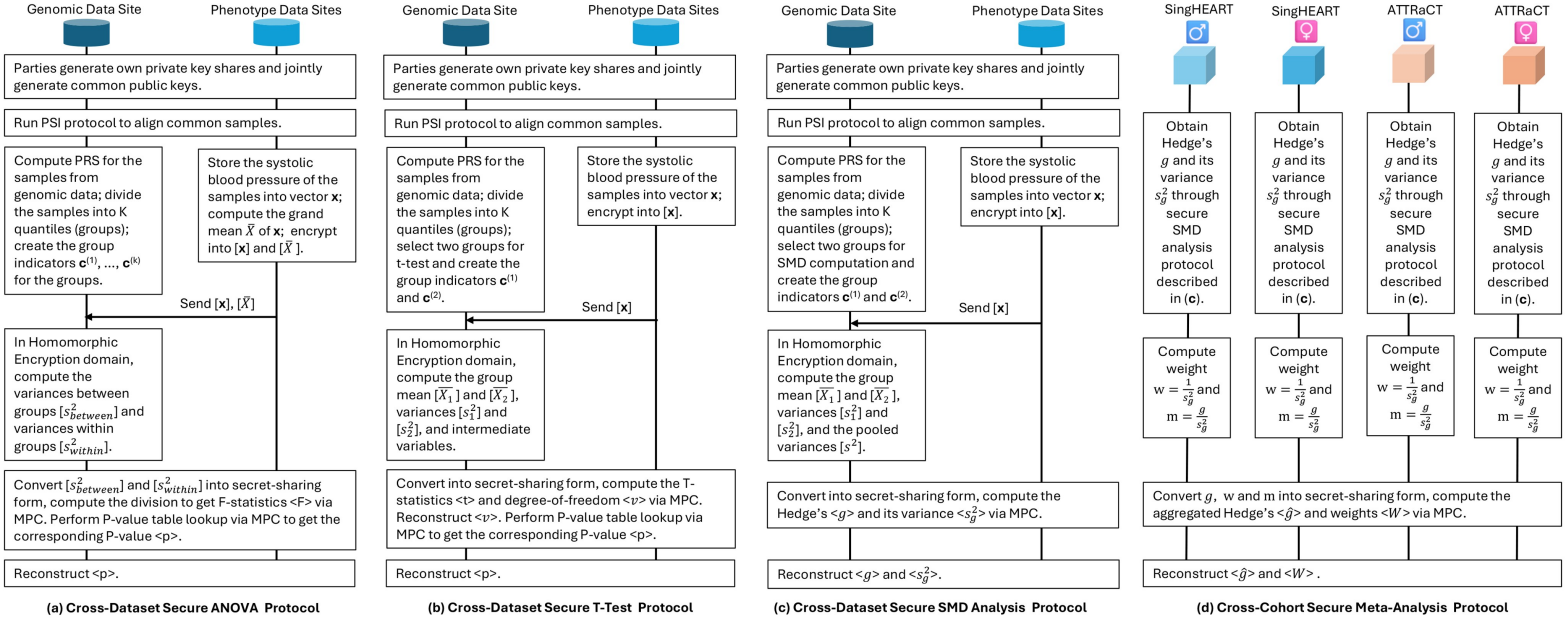
Protocols for the secure federated Welch’s *t*-test, ANOVA test, standardized mean difference (SMD) analysis and meta-analysis across private data sites. Welch’s *t*-test, ANOVA test, and SMD analysis are done across the genomic data site and phenotype data site within the SingHEART or ATTRaCT cohort respectively. Meta-analysis is done across the SingHEART and ATTRaCT cohorts. The number of phenotype data sites can be naturally extended to multiple participants under a hybrid data-partitioning scheme.

Our secure federated analysis platform implements the secure Welch’s *t*-test, secure one-way ANOVA test, and secure standardized mean difference (SMD) analysis across genomic data sites (providing PGS scores) and phenotype data sites (providing phenotypes such as gender, blood pressure), and secure meta-analysis on the SMD across different cohorts. The protocols for the secure federated analysis are described in Fig. 2.

#### Secure one-way analysis of variance (ANOVA)

The plain-text computation of the one-way ANOVA is well-known and implemented by various software packages; the key computation steps are listed in the supplementary data at *Bioinformatics* online. In the secure federated version, we consider the general case where one party own the grouping identifiers/classes of a set of participants (e.g. polygenic risk scores), and one or more parties own the attributes (e.g. systolic blood pressures) to be analyzed. To preserve the data privacy for all parties, only encrypted information will be exchanged between the parties; they will not disclose any raw plain-text data to each other. We describe below the design for the case of two parties, but note that this is extendable to the more general cases with three or more parties as well.

As shown in Fig. 2(a), there are two parties each holding the genomic dataset (Party 1) and phenotype dataset (Party 2) respectively. Party 2 possesses a vector of attribute values (e.g. systolic blood pressures) $x=[x_{1},\ldots ,x_{N}]$, with $x_{i}$ to be the attribute of sample *i*, and Party 1 computes *K* vectors of group indicators (e.g. PGS quintiles) $c^{\left(1\right)},\ldots ,c^{\left(K\right)}$ where $c^{\left(k\right)}=[c_{1}^{\left(k\right)},\ldots ,c_{N}^{\left(k\right)}]$ s.t. $c_{i}^{\left(k\right)}\in \{0,1\}$. In particular, if $c_{i}^{\left(k\right)}=1$, then the *i*-th sample $v_{i}$ belongs to group *k*; otherwise, $v_{i}$ does not belong to group *k*. The sample attribute values are private to Party 2, and the sample group information is private to Party 1. Only the total number of samples *N* and the total number of groups *K* are assumed to be publicly known to both parties. The secure ANOVA computation process is detailed in Algorithm 1, available as supplementary data at *Bioinformatics* online. In particular, *[[x]]* indicates *x* is encrypted as HE ciphertext, and 〈x〉 indicates *x* is in MPC secret-shared form. A function that begins with H or M signifies it is a HE or MPC primitive respectively. Conversion primitives like HE2MPC are explained in the previous section. The key design in our algorithm is that we leverage MHE to compute heavy linear computations efficiently through SIMD packing, while utilizing MPC to compute only lightweight nonlinear computations such as division which are generally difficult to compute in HE. Conversion is done between the two forms of computation without loss of security. In this way, our solution achieves optimal performance by leveraging the strength and overcoming the weakness of both cryptographic tools.

We utilize a private table look-up approach, denoted as MLookup, to obtain the nearest *P*-value from the corresponding pair of *F*-statistic (in secret-sharing form) and degree-of-freedom (in plain-text). We design the look-up table in such a way that the smaller the *P*-value is, the more fine-grained resolution the table has. In particular, we adopt a 0.001 interval for the range between 0 and 0.01, and 0.01 for the range between 0.01 and 0.1, and 0.1 for the range 0.1 to 1. This approach enables the secure ANOVA protocol to produce *P*-values with improved numerical precision in the most relevant range, especially near zero.

#### Secure Welch’s *t*-test

In Welch’s *t*-test, there are two groups to be compared, with corresponding sample sizes of $n_{1}$ and $n_{2}$. Similar to the ANOVA case, the key variables to compute securely are the *t*-statistic and degree of freedom, with which the corresponding *P*-value can be obtained from corresponding lookup tables for the *t*-distribution. The plain-text computational steps and the corresponding secure computation algorithm (Algorithm 2, available as supplementary data at *Bioinformatics* online) are described in the supplementary data at *Bioinformatics* online. The secure *t*-test protocol between the genomic data party and the phenotype data party is illustrated in Fig. 2(b).

#### Secure meta-analysis

Our secure platform implements the meta-analysis on SMD across SingHEART and ATTRaCT cohorts. For meta-analysis across multiple cohorts, each study cohort will conduct secure SMD analysis between its genomic and phenotype data parties first, then the analysis results from all the cohorts are aggregated to produce the meta-analysis results. For a given study cohort, let $\left\{n_{1},{\bar{X}}_{1},s_{1}\right\}$ and $\left\{n_{2},{\bar{X}}_{2},s_{2}\right\}$ be the sample sizes, means and standard deviations (e.g, systolic blood pressures) of groups 1 and 2 (e.g. quintiles of PGS) respectively. The pooled standard deviation *s* of the two groups is estimated as $\sqrt{\frac{s_{1}^{2}(n_{1}-1)+s_{2}^{2}(n_{2}-1)}{n_{1}+n_{2}-2}}$ and the SMD between the two groups is estimated by $\frac{{\bar{X}}_{1}-{\bar{X}}_{2}}{s}$ (a.k.a Cohen’s *d*). As the number of samples within groups may sometimes be small, bias correction is often done to give a corrected SMD estimator (Hedges’ *g*, Hedges 1981):


(1) $$g=\frac{{\bar{X}}_{1}-{\bar{X}}_{2}}{s}\times \left[1-\frac{3}{4(n_{1}+n_{2})-9}\right]$$

The estimator of the variance of Hedge’s *g* (Schlattmann 2009, Schwarzer *et al.* 2015, Borenstein *et al.* 2021) is:


(2) $$s_{g}^{2}=\frac{n_{1}+n_{2}}{n_{1}\times n_{2}}+\frac{g^{2}}{2(n_{1}+n_{2})-3.94}$$

The secure SMD protocol between the genomic data party and phenotype data party is shown in Fig. 2(c), and the detailed algorithm of the secure SMD analysis is described in Algorithm 3, available as supplementary data at *Bioinformatics* online.

For the meta-analysis of SMD across *R* studies, the pooled effect size (e.g. Hedges’ *g*) can be estimated under either a fixed-effect model, or a random-effect model. For the fixed-effect model, the aggregated effect size $\hat{g}$ is a weighted sum of the effect size $g_{r}$ from each study *r*, with the weight $w_{r}$ to be the inverse of the variance $s_{g_{r}}^{2}$ of the effect size:


(3) $$w_{r}=\frac{1}{s_{g_{r}}^{2}}$$

(4) $$\hat{g}=\frac{\sum_{r}\left(w_{r}\times g_{r}\right)}{\sum_{r}w_{r}}$$

The 95%-CI (confidence interval) for $\hat{g}$ can then be approximated as $\left[\hat{g}-1.96\sqrt{\frac{1}{\sum_{r}w_{r}}},\hat{g}+1.96\sqrt{\frac{1}{\sum_{r}w_{r}}}\right]$.

For the random-effect model, the between-study heterogeneity is quantified by the $\tau^{2}$ statistic and is added to the denominator of Equation 3. We use the DerSimonian and Laird (1986) estimator for $\tau^{2}$, which yields a closed-form expression based on Cochran’s *Q* statistic (Cochran 1954):


(5) $$Q=\sum_{r}\left(w_{r}\times g_{r}^{2}\right)-\frac{{\left(\sum_{r}w_{r}\times g_{r}\right)}^{2}}{\sum_{r}w_{r}}$$

(6) $$\tau^{2}=\frac{Q-(R-1)}{\sum_{r}w_{r}-\frac{\sum_{r}w_{r}^{2}}{\sum_{r}w_{r}}}$$

(7) $$w_{r}^{*}=\frac{1}{s_{g_{r}}^{2}+\tau^{2}}$$

The aggregated effect size ${\hat{g}}^{*}$ and corresponding confidence interval for random-effect model will follow the same procedures as in the fixed-effect model, by replacing the $w_{r}$ with $w_{r}^{*}$ in their respective formulations.

The protocol for secure meta-analysis across SingHEART and ATTRaCT cohorts is shown in Fig. 2(d). For each cohort, the secure SMD analysis is done on the male and female samples separately. The detailed algorithm for the general case of secure meta-analysis (fixed-effect model) across *R* studies is presented in Algorithm 4, available as supplementary data at *Bioinformatics* online. Our secure meta-analysis protocol ensures that the study-specific information is kept private within each cohort, and only the final pooled results are revealed to the requester. To validate the accuracy of our secure methodology, we performed the analogous meta-analysis on the plain-text using R v4.4.1 and the *meta* package (v7.0–0; Balduzzi *et al.* 2019) and generated the corresponding forest plots.

## Results

### Experimental settings

The testbed for our secure platform is deployed in a local-area network (LAN) environment with an average communication bandwidth of 1 Gbps. Each computation node is equipped with two 38-core Intel Xeon 8368Q CPUs and 512 GB of DRAM. Our MHE protocol is implemented using the OpenFHE library and is based on the CKKS (Cheon *et al.* 2017) scheme instantiated with standard parameters that provide 128-bit security, using a ring dimension of N=32,768 (logQ=730,logΔ=40). Under this configuration, the SIMD capability of CKKS enables the simultaneous processing of batches of size N/2=16,384. The MPC algorithms implemented in the MP-SPDZ framework using the SPDZ2K protocol configured in semi-honest mode with 40-bit statistical security.

### Evaluation on PGS for hypertension

#### Secure statistical tests

**Figure 3:**
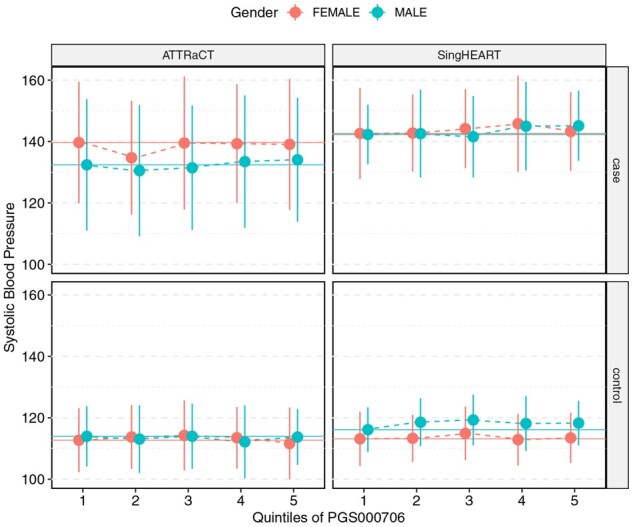
Systolic blood pressure (mmHg) of participants in the SingHEART and ATTRaCT cohorts vs quintiles of the polygenic risk score PGS000706, stratified by sex, case (with history of hypertension) and control (without history of hypertension).

**Figure 4:**
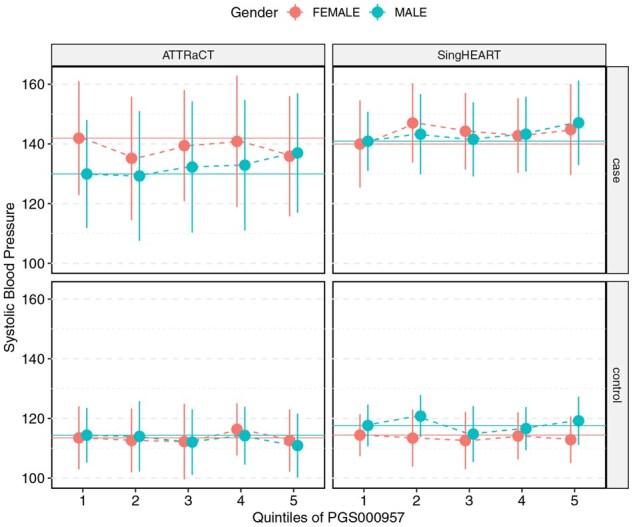
Systolic blood pressure (mmHg) of participants in the SingHEART and ATTRaCT cohorts vs quintiles of the polygenic risk score PGS000957, stratified by sex, case (with history of hypertension) and control (without history of hypertension).

**Figure 5:**
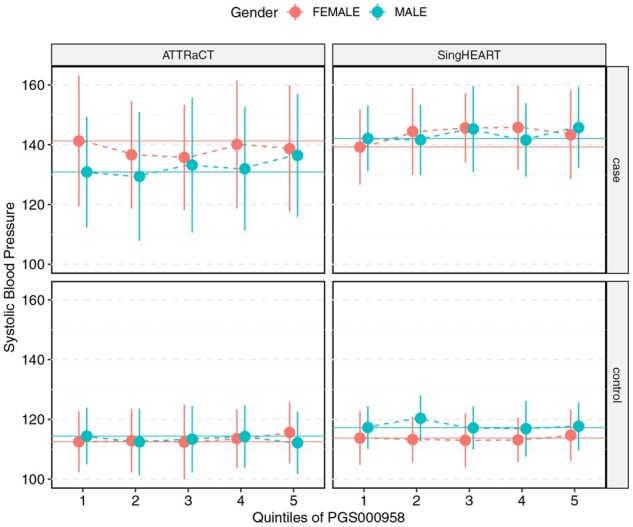
Systolic blood pressure (mmHg) of participants in the SingHEART and ATTRaCT cohorts vs quintiles of the polygenic risk score PGS000958, stratified by sex, case (with history of hypertension) and control (without history of hypertension).

The distributions of the systolic blood pressure of the participants between the five genetic risk groups are shown in Figs 3–5, separated by case/control and study. We note that the only necessary information to generate the figures is simply the mean and standard deviation of the respective subgroups/strata, which could be obtained securely from encrypted datasets via Algorithms 1 and 2, available as supplementary data at *Bioinformatics* online.

We compared the means between the five genetic risk groups for each study via ANOVA, and report the results (from both plain-text and encrypted implementations) in Table 1, available as supplementary data at *Bioinformatics* online. Similarly, we report the results of the *t*-tests conducted in Table 2, available as supplementary data at *Bioinformatics* online. Our secure version of these tests returned summary statistic values that are identical to the plain-text version. Differences in the returned *P*-value are consistent with the lookup table design described in the Methods section; we re-emphasize that the accuracy of the returned *P*-value can be arbitrarily achieved by increasing the resolution of the lookup table.

#### Secure meta-analysis

**Figure 6:**
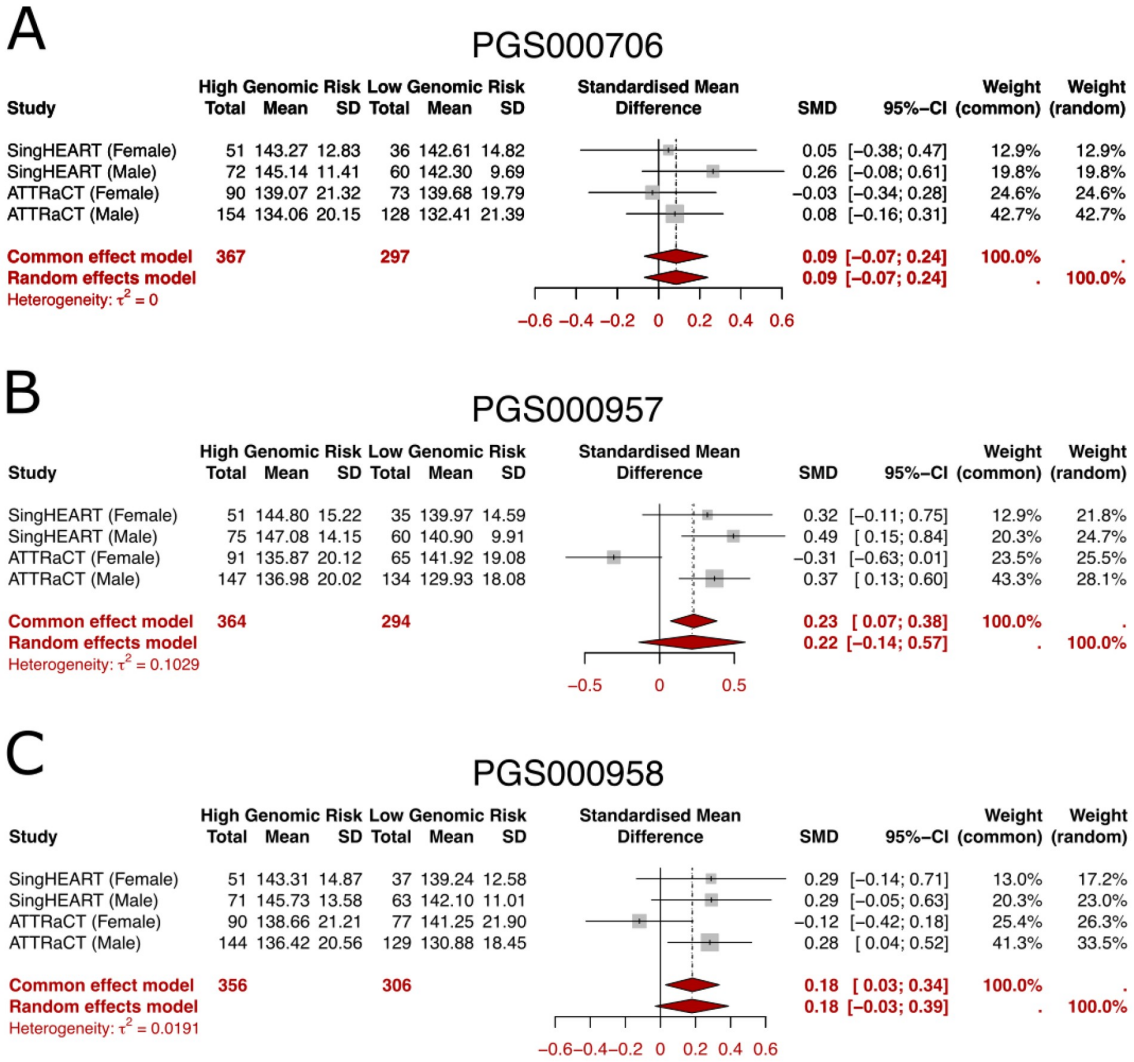
Forest plot for the meta-anaysis on the effect of genomic risk on systolic blood pressure (mmHg), for A: PGS000706; B: PGS000957; and C: PGS000958. For each cohort with specific sex, the Total, Mean, and SD refer to the group size, mean and variance on systolic blood pressure for the samples in the High Genomic Risk Group (e.g. fifth PGS quintile) and Low Genomic Risk Group (first PGS quintile) respectively. The results in red can be further computed in a privacy-preserving manner per the Methods section, so that meta-analysis of the standardized mean differences may proceed even if data owners do not wish to reveal cohort-level summary statistics (in black text).

We perform meta-analysis on the standardized mean difference (SMD) in systolic blood pressure between the highest and lowest genomic risk groups for each PRS under consideration. Figure 6 shows the intermediate statistics from each study (counts, mean and standard deviation), the corresponding SMDs, the τ-statistic for the random effect model, and the meta-analyzed SMD based on both common and random effects models. Note that the weights columns in the figures have been standardized to sum to 100%, i.e. the weight of cohort *i* would be $w_{i}/W$ per the notations used in the Methods section. The diamonds represent the pooled effects under the common and random effect models respectively, with the width of the diamonds being the 95%-confidence interval (Chang *et al.* 2022) of the pooled effect estimates. The intervals are only dependent on the computed term $s_{g}^{2}$ as per the Methods section. Although Fig. 6 was generated in R on the plain-text data, we verified that our secure implementation of the meta-analysis returned identical values for the pooled standardized mean differences (under both fixed and random effect models), confidence intervals and the $\tau^{2}$ statistic.

### Performance analysis

#### Comparison to pure MPC-based approach

As our solution orchestrates various secure protocols and switches between MPC and MHE (see Algorithms 1 and 2, available as supplementary data at *Bioinformatics* online) for various computations, we compared the runtime of our solution against a pure MPC solution. We computed a polygenic risk score for cholesterol levels (PGS000192) for participants in the 1000 Genomes project (Fairley *et al.* 2020), and performed *t*-tests on the scores for the following pairs using both solutions: (i) Americans vs East Asians (1075 participants), (ii) Europeans vs Africans (1526 participants), and (iii) Male vs Females (3202 participants).

**Figure 7:**
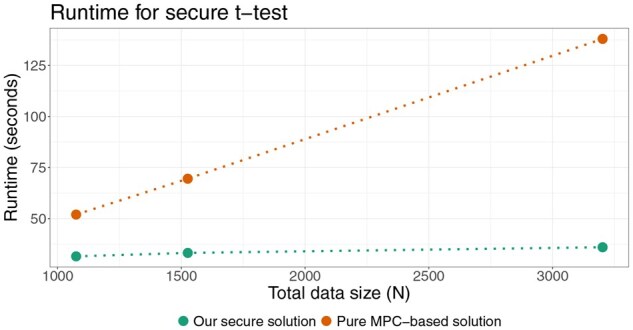
Comparison of runtimes of our secure t-test and a pure MPC-based solution.

The runtime performance of our secure platform against a pure MPC-based approach is shown in Fig. 7. We observed that our secure *t*-test solution had a constant runtime across the different data sizes presented (the largest being 3202 samples), whereas the runtime of the pure MPC-based solution had increased linearly.

#### Scalability across number of sites and sample sizes

To determine the impact of data size and number of parties on the runtime of our secure platform, we further conducted a series of experiments with simulated data. We varied the number of parties from 1 to 3, and the number of samples per party between 3000, 16 000 and 32 000. The largest configuration consisted of three parties each having 32 000 samples for a total of 96 000 samples. We performed secure ANOVA on each of these, and recorded the total runtime as well as the breakdown between the time spent on the MHE and MPC protocols.

**Figure 8:**
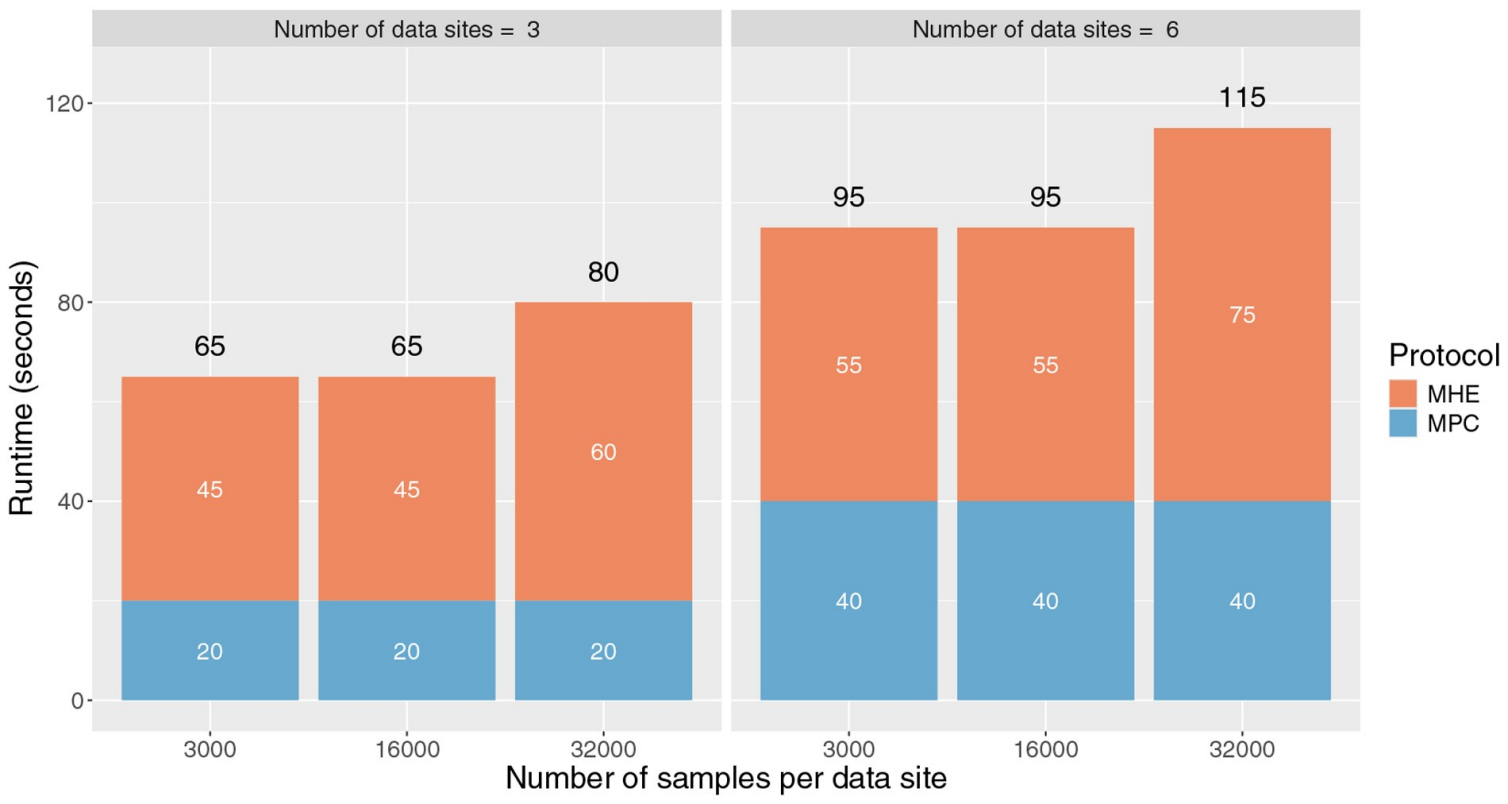
Scalability analysis on secure ANOVA runtimes by varying the number of samples per data site, and also the number of sites. The largest configuration here consists of a total of 6×32,000=192,000 samples. For each configuration, the total runtime is reported as the number above the resepctive bars. The runtime is further resolved into the the amount of time spent on MHE or MPC protocols (white text) inside our secure ANOVA solution.

Our secure ANOVA solution took 65 seconds to run on the smallest configuration (3×3000=9,000 total samples), and 115 seconds to run on the largest configuration (6×32,000=192,000 total samples). Despite the 20-fold increase in dataset size, the runtime increased by less than 2-fold. More generally, the runtime of secure ANOVA under these varying configurations (Fig. 8) reveal three important findings: (i) the total runtime of our secure solution increases only sublinearly with the total data size, (ii) the sublinear relationship between total data size and runtime is preserved even when the number of parties is doubled, (iii) a doubling in number of parties leads to an equivalent doubling in runtime of the MPC portions, but not on MHE portions. Due to the internal operations of the MHE protocol, increasing the dataset size does not result in noticeable runtime growth as long as the total data volume remains within the available MHE packing capacity. Once this capacity is exceeded, additional ciphertexts are required to accommodate the data, leading to a stepwise increase in runtime. Nevertheless, the overall MHE computation time grows sublinearly, as computations across multiple ciphertexts can be executed in parallel on multi-core CPUs.

## Discussions

### Related work on secure bioinformatics

In this work, we demonstrate that statistical analyses such as ANOVA and *t*-tests can be performed securely and scalably by integrating MHE with MPC. MHE efficiently handles linear computations via SIMD packing but is less suitable for non-linear operations such as division. MPC is more flexible for non-linear computations, yet incurs high communication overhead for large-scale linear operations. Our hybrid MHE+MPC approach significantly outperforms pure MPC approach and exhibits sublinear runtime growth in the MHE components with respect to dataset size. Moreover, total runtime scales sublinearly with the number of data sites, enabling support for large federated systems. Under the largest configuration tested—comparable in scale to the UK Biobank—our solution completed a secure ANOVA test in under two minutes.

Wirth *et al.* (2022) had previously provided a framework for performing *t*-test and ANOVA via repeated cycles of data summarization. However, we note that such methods expose the intermediate summary statistics (which could be sensitive to data owners), and their solution limits mathematical operations to additions and subtractions only. Dursi *et al.* (2021) has developed a federated data platform to enable collaborative research, but it relies on data access control policies aligned with the Global Alliance for Genomics and Health (GA4GH) guidelines. Froelicher *et al.* (2021) introduced MHE-based federated GWAS and Kaplan-Meier Survival Analysis, but their solution does not include an encryption conversion approach, and division operations are evaluated in approximated form via polynomials which may yield slower speed and lower accuracy. Their solution also computes complex functions such as the survival curve and *P*-value in plaintext.

In addition, other approaches to secure bioinformatics analysis include performing sensitive computations within trusted execution environments (TEE) or specialized secure hardware (Asvadishirehjini *et al.* 2020), as well as applying differential privacy (DP) mechanisms to reduce the risk of information leakage about individual participants (Zhou *et al.* 2022). TEE-based solutions typically offer strong performance advantages over pure cryptographic methods, as computations are performed on plaintext data within a hardware-protected enclave. However, their security guarantees rely on assumptions about hardware trust and resistance to side-channel or implementation-level attacks. In contrast, DP offers quantifiable privacy guarantees by introducing carefully calibrated noise into the analysis. However, this typically results in reduced analytical accuracy, and it also does not fully eliminate the risk of information leakage, unlike cryptographic methods such as MHE and MPC. Therefore, the use of these methods in real-world settings is often limited, particularly in highly sensitive domains such as bioinformatics.

### Biological findings

Although the focus of this work is on demonstrating secure collaborative analytics, our investigation of the polygenic risk scores from the ATTRaCT dataset had not been previously published. Our results from the polygenic score evaluation are consistent with findings from other studies (Duncan *et al.* 2019, Martin *et al.* 2019) that scores developed on European-majority populations do not generalize well in populations with different demographics, such as those from an Asian country like Singapore. Amongst the three polygenic risk scores, PGS000957 and PGS000958 showed a clearer relationship between higher risk categories (based on quintiles of underlying scores) and the actual measured systolic blood pressure of hypertension cases. This is particularly true for males in the SingHEART and ATTRaCT cohorts, where the meta-analysis showed consistently positive standardized mean differences for those subsets. Although it is more common in meta-analysis literature to conduct a subgroup analysis separately (i.e. study the effects of males and females separately here), we note that the respective polygenic risk scores were not sex-specific and are not expected to show significant differences between the two sexes.

## Conclusion

Federated analytics across multiple study cohorts and data sites can be conducted in a secure manner using privacy-preserving techniques such as multiparty homomorphic encryption. We present a scalable solution for statistical analysis, and posit that such methods will underpin future data sharing models in the biomedical space, replacing the need for cumbersome co-localized data repositories.

## Supplementary Material

btag081_Supplementary_Data

## Data Availability

The data underlying this article were provided by the National Heart Centre Singapore (NHCS) through the SingHEART and Asian neTwork for Translational Research and Cardiovascular Trials (ATTRaCT) research platforms. The data cannot be shared publicly due to ethical and privacy restrictions under the Singapore Personal Data Protection Act, as they contain sensitive clinical, genomic, and cardiac imaging information. De-identified data may be made available to qualified researchers upon reasonable request, subject to the approval of the SingHealth/NHCS Centralized Institutional Review Board and the respective cohort Steering Committees. Requests for the access to the datasets should be directed to the respective co-author: SingHEART Contact: Prof Khung Keong Yeo (yeo.khung.keong@singhealth.com.sg) ATTRaCT Contact: Prof Roger Foo (roger.foo@nus.edu.sg)
