# Supplementary material for: Secure bioinformatics: privacy-preserving federated analytics using homomorphic encryption
Source: Bioinformatics. 2026 Feb 20;42(5):btag081. doi: 10.1093/bioinformatics/btag081 (PMC13157224; doi:10.1093/bioinformatics/btag081)
Supplement: btag081_Supplementary_Data [file btag081_supplementary_data.pdf]

Acknowledgement for ATTRaCT cohort

Prof. Patrick Cozzone, Dr. Han Weiping, Dr. Phillip Lee, Prof. Stuart Cook, Assoc. Prof. Tan Ru San, Prof. David Townsend, Dr. John Totman, Prof. Peter Little, Prof. Dominique de Kleijn, Prof. Colin Stewart, Prof. Roger Foo, Prof. Ng Huck Hui, Prof. JJ Liu, Prof. Mark Richards, Dr. Anis Larbi, Dr. Brian Abel, Dr. Alessandra Nardin, Dr. Michael Poidinger, Prof. Laurent Renia, Assoc. Prof. Jayantha Gunaratne, Prof. Salvatore Albani, Prof. Theodoros Kofidis, Dr. Sorokin Vitaly, Dr. Jai Ajitchandra Sule, Dr. Guohao Chang, Dr. Huang Wenjie, Dr. Deanna Khoo, Dr. Tong Jieli, Dr. Poh Shaun Daniel Yeo, Dr. Thet Su Su, Dr. Tun Thet, Dr. Colin Yeo, Dr. Ong Hean Yee, Dr. Yap Siew Fei, Assoc. Prof Tan Ru San, Dr. Angela Koh Su-Mei, Dr. David Sim Kheng Leng, Dr. Tang Hak Chiaw, Prof. Derek J Hausenloy, Dr. Louis Teo Loon Yee, Prof. Carolyn Lam Su Ping, Dr. Catherine Dong Yan Hong, Assoc. Prof. Ling Lieng His, Assoc. Prof. Ng Tze Pin, Dr. Qin Yan, Dr. Haresh Tulsidas, Dr. Ching Min Er, Dr. Calvin Chin Woon Loong, Dr. Gerard Leong Kui Toh, Assoc. Prof. Jaufeerally Fazlur Rehman, Jean-Paul Kovalik, Khoo Chin Meng, Dr. Loh Seet Yoong, Dr. Sheldon Lee Shao Guang, Dr. Dinna Soon, Dr. Aguite Leizal, Dr. Chew Chai Siang, Dr. Huang Wei Liang, Dr. Liew Boon Wah, Dr. Macdonald Michael Ross, Dr. Mancenido Amelle M E, Dr. Rathees Murugesapillai, Dr. Tan Vern Hsen, Dr. Jasper Tromp, Dr. Wouter Ouwerkerk, Dr. Jin Xuanyi, Dr. Lynette Teo Li San, Dr. Raymond C Wong, Dr. Kar Yin Se, Dr. Ping Chai, Assoc. Prof Kian Keong Poh, Dr. Lee Fong Ling, Dr. Yong Quekwei, Chang Fen Xu.

Competing interests

Carolyn SP Lam is supported by a Clinician Scientist Award from the National Medical Research Council of Singapore; has Received research support from NovoNordisk and Roche Diagnostics; has Served as consultant or on the Advisory Board/ Steering Committee/ Executive Committee for Alleviant Medical, Allysta Pharma, Alnylam Pharma, AnaCardio AB, Applied Therapeutics, AstraZeneca, Bayer, Biopeutics, Boehringer Ingelheim, Boston Scientific, Bristol Myers Squibb, CardioRenal, CPC Clinical Research, Eli Lilly, Hanmi, Impulse Dynamics, Intellia Therapeutics, Ionis Pharmaceutical, Janssen Research & Development LLC, Medscape/WebMD Global LLC, Merck, Novartis, Novo Nordisk, Prosciento Inc, Quidel Corporation, Radcliffe Group Ltd., Recardio Inc, ReCor Medical, Roche, Sanofi, Siemens Healthcare Diagnostics and Us2.ai; and serves as Co-founder & non-executive director of Us2.ai.

Acknowledgments

This work is supported in part by the National Research Foundation, Singapore and Infocomm Media Development Authority under its Trust Tech Funding Initiative (DTC-RGC-01), the A*STAR Council Strategic Fund (CSF-SERC: C210415010) and the National Precision Medicine Phase I (IAF-PP: H17/01/a0/007). Any opinions, findings and conclusions or recommendations expressed in this material are those of the author(s) and do not reflect the views of National Research Foundation, Singapore and Infocomm Media Development Authority.

We will like to thank the Lee Foundation for grant support to the SingHEART study conducted at the National Heart Centre Singapore. SingHEART study also received grant support in memory of Mr Henry H L Kwee. This work was also supported by core funding from SingHealth and Duke-NUS Institute of Precision Medicine (PRISM) and the centre grant awarded to the National Heart Centre Singapore from the National Medical Research Council, Ministry of Health, Singapore (NMRC/CG/M006/2017_NHCS and MOH-000985).

We wish to acknowledge The Asian neTwork for Translational Research and Cardiovascular Trials (ATTRaCT) for sharing the data to perform this work. Acknowledgement of all key researchers involved in the ATTRaCT cohort is provided in the supplementary. ATTRaCT is supported by the following grants: NMRC grant – CVRI Centre Grant (NMRC/CG/014/2013), NRMC grant - NMRC Translational and Clinical Research (TCR) Flagship Programme Tier 1 (NMRC/TCR/006-NUHS/2013) and the A*STAR BMRC – Strategic Positioning Fund (SPF) grant (Reference number SPF2014/003; SPF2014/004; SPF2014/005).

We also thank the BiomedDAR and team, particularly Wong Wing Cheong and Max Fun, for their assistance in setting up the infrastructure. The infrastructure is supported by Joint Platform 1 under the CArdiovascular DiseasE National Collaborative Enterprise (CADENCE) National Clinical Translational Program (MOH-001277-01).

One-way Analysis of Variance (ANOVA)

Suppose there are a total of N samples, coming from K groups to be compared. We denote the sample size for group i as n_i. Then the key computation steps for an ANOVA performed in plain-text form are:

1. Calculating the mean of each group ($\bar{X}_k$)
2. Calculating the grand mean ($\bar{X}$)
3. Calculating "variance between groups":

$$s^2_{between} = \frac{\sum_{i=1}^{K} n_i(\bar{X}_i - \bar{X})^2}{K-1} \tag{1}$$

4. Calculating "variance within groups":

$$s^2_{within} = \frac{\sum_{j=1}^{K} \sum_{i=1}^{n_j} (x_{ij} - \bar{X}_j)^2}{N-K} \tag{2}$$

5. Calculating F-ratio:

$$F = \frac{s^2_{between}}{s^2_{within}} \tag{3}$$

The denominators in equations 1 and 2 are known as the degree of freedoms for "between group" and "within group" respectively. The F-ratio and the two degree of freedoms are used jointly to get the corresponding p-values.

Welch's t-test

The main computation steps for the test are as follows:

1. Calculating the mean of each group ($\bar{X}_i$)
2. Calculating the standard deviation of each group (s_i)
3. Calculating t-statistic:

$$t = \frac{\bar{X}_1 - \bar{X}_2}{\sqrt{\frac{s_1^2}{n_1} + \frac{s_2^2}{n_2}}} \tag{4}$$

4. Calculating the degree of freedom (ν):

$$\nu = \frac{\left(\frac{s_1^2}{n_1} + \frac{s_2^2}{n_2}\right)^2}{\frac{s_1^4}{n_1^2(n_1-1)} + \frac{s_2^4}{n_2^2(n_2-1)}} \tag{5}$$

Algorithms

Algorithm 1: Secure ANOVA between two clients

Public input: N, K
Private input: Client 1: $\mathbf{x}$; Client 2: $\mathbf{c}^{(1)}, ..., \mathbf{c}^{(K)}$, and $n_1, ..., n_K$
Result: p that approximates the p-value of the ANOVA test

Client 1
  Compute grand mean $\bar{X} \leftarrow \frac{\sum \mathbf{x}}{N}$;
  Replicate and encrypt $\bar{X}$ into $[\![\bar{X}]\!]$;
  Encrypt $\mathbf{x}$ and send $[\![\mathbf{x}]\!]$ and $[\![\bar{X}]\!]$ to Client 2;
end
Client 2
  for $k \leftarrow 1; k \leq K; k \leftarrow k+1$ **do**
    $[\![\bar{X}_k]\!] \leftarrow$ HSlotSum(HMult($[\![\mathbf{x}]\!]$, $\frac{\mathbf{c}^{(k)}}{n_k}$));
    $[\![a]\!] \leftarrow$ HSquare(HSub($[\![\bar{X}_k]\!]$, $[\![\bar{X}]\!]$));
    $[\![a]\!] \leftarrow$ HMult($[\![a]\!]$, $\frac{n_k}{K-1}$);
    $[\![s^2_{between}]\!] \leftarrow$ HAdd($[\![s^2_{between}]\!]$, $[\![a]\!]$);
    $[\![b]\!] \leftarrow$ HMult(HSub($[\![\mathbf{x}]\!]$, $[\![\bar{X}_k]\!]$), $\mathbf{c}^{(k)}$);
    $[\![b]\!] \leftarrow$ HSlotSum(HSquare($[\![b]\!]$));
    $[\![s^2_{within}]\!] \leftarrow$ HAdd($[\![s^2_{within}]\!]$, $[\![b]\!]$);
  end
  $[\![s^2_{within}]\!] \leftarrow$ HMult($[\![s^2_{within}]\!]$, $\frac{1}{N-K}$);
end
Client 1 and 2
  $\langle s^2_{between} \rangle \leftarrow$ HE2MPC($[\![s^2_{between}]\!]$);
  $\langle s^2_{within} \rangle \leftarrow$ HE2MPC($[\![s^2_{within}]\!]$);
  $\langle F \rangle \leftarrow$ MDiv($\langle s^2_{between} \rangle$, $\langle s^2_{within} \rangle$);
  $\langle p \rangle \leftarrow$MLookup(P_Table, $\langle F \rangle, N-K, K-1$);
  Reveal p;
end
return p;

Algorithm 2: Secure t-test between two clients

Public input: None
Private input: Client 1: $\mathbf{x}$; Client 2: $\mathbf{c}^{(1)}, \mathbf{c}^{(2)}$, and n_1, n_2
Result: p that approximates the p-value of the t-test

Client 1
  Encrypt $\mathbf{x}$ and send $[\![\mathbf{x}]\!]$ to Client 2;
end
Client 2
  $[\![\bar{X}_1]\!] \leftarrow$ HSlotSum(HMult($[\![\mathbf{x}]\!]$, $\frac{\mathbf{c}^{(1)}}{n_1}$));
  $[\![\bar{X}_2]\!] \leftarrow$ HSlotSum(HMult($[\![\mathbf{x}]\!]$, $\frac{\mathbf{c}^{(2)}}{n_2}$));
  $[\![e_1]\!] \leftarrow$ HSquare(HSub($[\![\bar{X}_1]\!]$, $[\![\mathbf{x}]\!]$));
  $[\![s^2_1]\!] \leftarrow$ HSlotSum(HMult($[\![e_1]\!]$, $\frac{\mathbf{c}^{(1)}}{n_1-1}$)));
  $[\![e_2]\!] \leftarrow$ HSquare(HSub($[\![\bar{X}_2]\!]$, $[\![\mathbf{x}]\!]$));
  $[\![s^2_2]\!] \leftarrow$ HSlotSum(HMult($[\![e_2]\!]$, $\frac{\mathbf{c}^{(2)}}{n_2-1}$));
  $[\![t^2_{nu}]\!] \leftarrow$ HSqure(HSub($[\![\bar{X}_1]\!]$, $[\![\bar{X}_2]\!]$));
  $[\![t^2_{de}]\!] \leftarrow$ HAdd(HMult($[\![s^2_1]\!]$, $\frac{1}{n_1}$), HMult($[\![s^2_2]\!]$, $\frac{1}{n_2}$));
  $[\![\nu_{nu}]\!] \leftarrow$ HSquare($[\![t^2_{de}]\!]$);
  $[\![\nu_{de}]\!] \leftarrow$ HAdd(HMult(HSquare($[\![s^2_1]\!]$), $\frac{1}{n_1^2(n_1-1)}$), HMult(HSquare($[\![s^2_2]\!]$), $\frac{1}{n_2^2(n_2-1)}$));
end
Client 1 and 2
  $\langle t^2_{nu} \rangle \leftarrow$ HE2MPC($[\![t^2_{nu}]\!]$);
  $\langle t^2_{de} \rangle \leftarrow$ HE2MPC($[\![t^2_{de}]\!]$);
  $\langle t^2 \rangle \leftarrow$ MDiv($\langle t^2_{nu} \rangle$, $\langle t^2_{de} \rangle$);
  $\langle \nu_{nu} \rangle \leftarrow$ HE2MPC($[\![\nu_{nu}]\!]$);
  $\langle \nu_{de} \rangle \leftarrow$ HE2MPC($[\![\nu_{de}]\!]$);
  $\langle \nu \rangle \leftarrow$ MDiv($\langle \nu_{nu} \rangle$, $\langle \nu_{de} \rangle$);
  Reveal ν;
  $\langle p \rangle \leftarrow$MLookup(P_Table, $\langle t^2 \rangle, \nu$);
  Reveal p;
end
return p;

Algorithm 3: Secure SMD analysis for each study

Public input: None
Private input: Client 1: $\mathbf{x}$; Client 2: $\mathbf{c}^{(1)}, \mathbf{c}^{(2)}$, and n_1, n_2
Result: Standard mean difference g and variance s_g^2

Client 1
- Encrypt $\mathbf{x}$ and send $[\![\mathbf{x}]\!]$ to Client 2;

end
Client 2
- $[\![\bar{X}_1]\!] \leftarrow$ HSlotSum(HMult($[\![\mathbf{x}]\!], \frac{\mathbf{c}^{(1)}}{n_1}$));
- $[\![\bar{X}_2]\!] \leftarrow$ HSlotSum(HMult($[\![\mathbf{x}]\!], \frac{\mathbf{c}^{(2)}}{n_2}$));
- $[\![e_1]\!] \leftarrow$ HSquare(HSub($[\![\bar{X}_1]\!], [\![\mathbf{x}]\!]$));
- $[\![s_1^2]\!] \leftarrow$ HSlotSum(HMult($[\![e_1]\!], \frac{\mathbf{c}^{(1)}}{n_1-1}$)));
- $[\![e_2]\!] \leftarrow$ HSquare(HSub($[\![\bar{X}_2]\!], [\![\mathbf{x}]\!]$));
- $[\![s_2^2]\!] \leftarrow$ HSlotSum(HMult($[\![e_2]\!], \frac{\mathbf{c}^{(2)}}{n_2-1}$));
- $[\![s^2]\!] \leftarrow$ HAdd(HMult($[\![s_1^2]\!], \frac{n_1-1}{n_1+n_2-2}$),HMult($[\![s_2^2]\!], \frac{n_2-1}{n_1+n_2-2}$));
- $[\![d^2]\!] \leftarrow$ HSqure(HSub($[\![\bar{X}_1]\!], [\![\bar{X}_2]\!]$));

end
Client 1 and 2
- $\langle d^2 \rangle \leftarrow$ HE2MPC($[\![d^2]\!]$);
- $\langle s^2 \rangle \leftarrow$ HE2MPC($[\![s^2]\!]$);
- $\langle g^2 \rangle \leftarrow$ MDiv($\langle d^2 \rangle, \langle s^2 \rangle$);
- Reveal g^2;

end
Client 2
- $g \leftarrow g \times (1 - \frac{3}{4(n_1+n_2)-9})$;
- $s_g^2 = \frac{n_1+n_2}{n_1 \times n_2} + \frac{g^2}{2(n_1+n_2)-3.94}$;

end
return g, s_g^2;

Algorithm 4: Secure meta analysis across R studies

Public input: None
Private input: For each study r: g_r and $s_{g_r}^2$
Result: Aggregated $\hat{g}$ and weights W

Study r
- ▽ `Convert inputs into secret-shared data`
- $\langle g_r \rangle \leftarrow g_r$;
- $\langle w_r \rangle \leftarrow \frac{1}{s_{g_r}^2}$;
- $\langle m_r \rangle \leftarrow \frac{g_r}{s_{g_r}^2}$;

end
Study 1 ... R
- $\langle W \rangle \leftarrow$ MAdd($\langle w_1 \rangle, \ldots, \langle w_R \rangle$);
- Reveal W;
- $\langle M \rangle \leftarrow$ MAdd($\langle m_1 \rangle, \ldots, \langle m_R \rangle$);
- $\langle \hat{g} \rangle \leftarrow$ MMult($\langle M \rangle, \frac{1}{W}$);
- Reveal $\hat{g}$;

end
return $\hat{g}, W$;

Additional Results

Table 1. Results of ANOVA F-test on hypertensive cases in the two study cohorts

PGS	Study Cohort	Sex	Plain-text F-statistic	Plain-text p-value	Encrypted F-statistic	Encrypted p-value
PGS000706	SingHEART	Female	0.418	0.796	0.418	0.8
		Male	1.13	0.342	1.13	0.4
	ATTRaCT	Female	0.745	0.562	0.745	0.6
		Male	0.701	0.592	0.701	0.6
PGS000957	SingHEART	Female	1.30	0.270	1.30	0.3
		Male	2.54	0.0400	2.54	0.04
	ATTRaCT	Female	1.66	0.158	1.66	0.2
		Male	2.98	0.0188	2.98	0.02
PGS000958	SingHEART	Female	1.52	0.197	1.52	0.2
		Male	1.79	0.130	1.79	0.2
	ATTRaCT	Female	0.985	0.415	0.985	0.5
		Male	2.33	0.0545	2.33	0.06

For each polygenic score, the mean systolic blood pressures of individuals from the five genetic risk groups were compared via the ANOVA F-test. The tests were done separately for each study cohort, and stratified by gender. Quantitative values of the F-statistic and p-value resulting from the ANOVA tests in both the plain-text version and secure methodology (encrypted) are reported in the table.

Table 2. p-values from t-test between selected genetic risk (GR[1]) groups in hypertensive cases

PGS	Study Cohort	Sex	Plain-text GR 5 vs GR 1	Plain-text GR 5 vs GR 3	Encrypted GR 5 vs GR 1	Encrypted GR 5 vs GR 3
PGS000706	SingHEART	Female	0.829	0.725	0.9	0.8
		Male	0.125	0.0877	0.2	0.09
	ATTRaCT	Female	0.848	0.893	0.9	0.9
		Male	0.51	0.275	0.6	0.3
PGS000957	SingHEART	Female	0.142	0.835	0.2	0.9
		Male	0.00345	0.0138	0.004	0.02
	ATTRaCT	Female	0.0581	0.225	0.06	0.3
		Male	0.00213	0.0509	0.003	0.06
PGS000958	SingHEART	Female	0.169	0.395	0.2	0.4
		Male	0.0896	0.843	0.09	0.9
	ATTRaCT	Female	0.441	0.336	0.5	0.4
		Male	0.0199	0.202	0.02	0.3

Unadjusted p-values resulting from t-tests between selected genetic risk groups.

[1]GR 5 is the highest genetic risk group, while GR 1 is the lowest genetic risk group. GR 3 represents the average genetic risk group.
